# The chain mediating role of emotion dysregulation and non-suicidal self-injury in the relationship between cyber-victimization and suicidal ideation among college students

**DOI:** 10.1080/00049530.2026.2644951

**Published:** 2026-04-08

**Authors:** Meng Gao, Chunfen Zhou, Jing Diao, Chen Liu, Xinxin Zhang, Haixing Wang, Hongmei Liu, Ying Lei

**Affiliations:** a Mental Health Education Center, University of Electronic Science and Technology of China, Chengdu, China; b Mental Health Center, West China Hospital, Sichuan University/West China School of Nursing, Chengdu, China

**Keywords:** Emotion dysregulation, suicidal ideation, non-suicidal self-injury, cyber-victimization, college students

## Abstract

**Objective:**

Cyber-victimization has been increasingly linked to suicidal ideation, yet the underlying psychological mechanisms remain underexplored among college students. This study examined the relationship between cyber-victimization and suicidal ideation among Chinese college students, with a focus on the mediating roles of emotion dysregulation and non-suicidal self-injury.

**Method:**

A total of 2001 students aged 18–24 completed questionnaires. Common method bias tests, descriptive statistics, Pearson correlation analyses, and chain mediation analysis (using the PROCESS Model 6) were conducted.

**Results:**

Suicidal ideation was affected by cyber-victimization through 3 different pathways: the mediating role of emotion dysregulation, the mediating role of non-suicidal self-injury, and the chain mediating role of both emotion dysregulation and non-suicidal self-injury.

**Conclusions:**

This study demonstrates that emotion dysregulation and non-suicidal self-injury sequentially mediate the relationship between cyber-victimization and suicidal ideation among college students, underscoring the need for early interventions to strengthen emotion regulation and reduce non-suicidal self-injury.

## KEY POINTS

**What is already known about this topic:**

1. Cyber-victimization is positively associated with suicidal ideation.
2. Cyber-victimized individuals are 15 times more likely to have a history of non-suicidal self-injury.
3. Little is known about the mechanisms underlying the impact of cyber-victimization on suicidal ideation among college students.

**What this topic adds:**

1. Cyber-victimization is indirectly associated with suicidal ideation through the mediating effects of emotion dysregulation and non-suicidal self-injury.
2. Emotion dysregulation and engagement in non-suicidal self-injury exacerbate the risk of suicidal ideation associated with cyber-victimization.
3. Little is known about how competent Australian MHPs are to provide cross lingual psychotherapy nor what predicts cross-lingual competence.

## Introduction

Suicide remains a critical public health issue globally, especially among young people. Data from the World Health Organization (2025) estimates that over 727,000 individuals died by suicide worldwide in 2021, and suicide ranks as the third leading cause of death following road injuries and interpersonal violence among people aged 15–29. In this age group, college students attract significant attention to their mental health and suicide. A previous study showed that suicide is the foremost cause of unnatural death among college students in China, with 47.2% of the unnatural death population (Yang & Li, 2015). Every suicide highlights the fact that many more individuals attempt suicide and experience recurring suicidal ideation. Suicidal ideation has been considered an important precursor to suicide (Zhao et al., 2025) and serves as the strongest predictor of suicidal behaviour (Miranda et al., 2014). Suicidal ideation refers to a cognitive process in which individuals think about, consider, or plan to end their own life without any actions to suicide (Klonsky et al., 2016), as the first step to suicidal attempt and suicidal behaviour. Research with a sample of 84,850 adults across 17 countries found a lifetime prevalence of suicidal ideation at 9.2% (Nock et al., 2008). Given these statistics, it is crucial to identify the risk factors that contribute to the formation and development of suicidal ideation among college students, as well as the protective factors that can aid in prevention and treatment.

The proliferation of electronic media has garnered significant attention regarding the detrimental effects of cyber-victimization (Zou et al., 2023). Cyber-victimization refers to the recurrent dissemination of hostile or offensive messages by an individual or group via electronic or digital media, with the intent to inflict harm or discomfort upon others (Tokunaga, 2010). In contrast to traditional bullying victimization, cyber-victimization is marked by anonymity, extensive accessibility, rapid dissemination of harmful content, and the potential for enduring psychological effects. These characteristics may intensify the psychological distress experienced by victims. Researchers maintain that the college years are a crucial developmental period, representing the transition from adolescence to adulthood, often referred to as emerging adulthood (Arnett et al., 2014). During this phase, students transition from living under parental supervision to independent living, necessitating adjustments in areas such as survival skills, socialisation, and emotional regulation (Ferrer-Cascales et al., 2019). This transition increases the internet exposure and, consequently, vulnerability to online harassment. Recent empirical investigations have further delineated the prevalence and impact of cyber-victimization among Chinese college students. For instance, a study of 8098 Chinese college students identified a 12-month prevalence rate of cyber-victimization at 7.82% (Jin et al., 2023). Another large-scale study, comprising 18,578 Chinese college students, revealed that 10.1% of participants had experienced cyber-victimization (Sun et al., 2024). Several studies indicate that cyber-victimization is positively associated with suicidal ideation (Chu et al., 2022; Peng et al., 2019; Zou et al., 2023). However, there is little information about the precise mechanisms between cyber-victimization and suicidal ideation.

One widely accepted perspective on suicide posits that psychological pain plays a crucial role in the emergence of suicidal ideation (Baumeister, 1990; Klonsky & May, 2015; Shneidman, 1993). The three-step theory of suicide proposed by Klonsky and May (2015) provides a comprehensive framework for understanding the progression from suicidal ideation to actions. Suicidal ideation often arises when individuals experience persistent and intolerable psychological pain that they struggle to manage. This pain can escalate, especially if they feel disconnected from others, and ultimately may develop into suicidal behaviour. Therefore, individuals who struggle with emotional dysregulation and cannot cope with psychological pain may begin to experience suicidal ideation (Turton et al., 2021). Gratz and Roemer (2004) define emotion regulation as the awareness, acceptance, and understanding of emotions, along with the capacity to manage impulsive behaviours and apply appropriate strategies based on the situation and personal goals. When adaptive strategies (e.g., cognitive reappraisal) are insufficient or unavailable, individuals may resort to maladaptive strategies (e.g., expressive suppression), resulting in heightened and prolonged psychological pain. Another research suggests that cyber-victims suppress their emotions significantly more than other groups (Vranjes et al., 2018). This suggests that those who experience cyber-victimization are more likely to face emotional dysregulation. Consequently, we hypothesise that emotional dysregulation mediates the relationship between cyber-victimization and suicidal ideation.

Additionally, it is worth noting that NSSI also plays a role in predicting suicidal ideation among college students. Non-suicidal self-injury (NSSI) is a maladaptive response to intense negative emotions, which is defined as the deliberate destruction of bodily tissue without suicidal intent (Nock, 2010). Research including 6393 college students assessing the associations between past NSSI and future suicidal ideation showed that NSSI was associated with increased subsequent suicide ideation (Kiekens et al., 2018). A longitudinal study demonstrated that each additional point in reported NSSI at baseline was associated with more than a fivefold increase in the odds of a future occurrence of elevated suicide ideation (Guan et al., 2012). Studies have found that cyber-victimization is a significant risk factor for NSSI. Two cross-sectional surveys exploring the relationship between cyber-victimization and NSSI among college students indicate a positive association (Liao et al., 2025; Liu et al., 2025). Individuals who have experienced cyber-victimization are approximately 15 times more likely to report a history of NSSI compared to those who have not faced such experiences (Lanzillo et al., 2023). A few studies have focused on the relationship between cyber-victimization, NSSI, and suicidal ideation among adolescents (Lanzillo et al., 2023; Peng et al., 2019), while little is known about all three of them among college students. Liu et al. (2024) found that cyber-victimization exhibited unidirectional positive cross-lagged effects on NSSI, and NSSI could subsequently indirectly be associated with suicidal ideation. Therefore, we hypothesise that NSSI mediates the relationship between cyber-victimization and suicidal ideation.

The experiential avoidance model suggests that engagement in self-injury is primarily maintained by negative reinforcement, in the form of escape from or avoidance of unwanted emotional experiences (Chapman et al., 2006). From this perspective, NSSI functions as a short-term emotion regulation strategy that temporarily alleviates distress but reinforces maladaptive coping over time. Individuals who struggle to regulate overwhelming emotions may adopt self-injury as a maladaptive yet immediate emotion-regulation strategy. Klonsky (2007) emphasised that emotion regulation is one of the most robustly supported functions of NSSI. Meta-analytic evidence further confirms that emotion dysregulation is one of the strongest psychological correlates of NSSI (Wolff et al., 2019). Longitudinal evidence suggests that peer victimization predicts subsequent increases in emotion regulation difficulties, which, in turn, are associated with later self-injurious behaviours (McLaughlin et al., 2011). Taken together, these findings suggest a potential sequential pathway in which emotion dysregulation may precede and increase vulnerability to NSSI. Thus, we propose that emotion dysregulation represents a proximal psychological mechanism triggered by cyber-victimization, which in turn increases the likelihood of engaging in NSSI as a maladaptive emotion regulation strategy, ultimately elevating the risk of suicidal ideation.

## The present study

**Figure 1.**
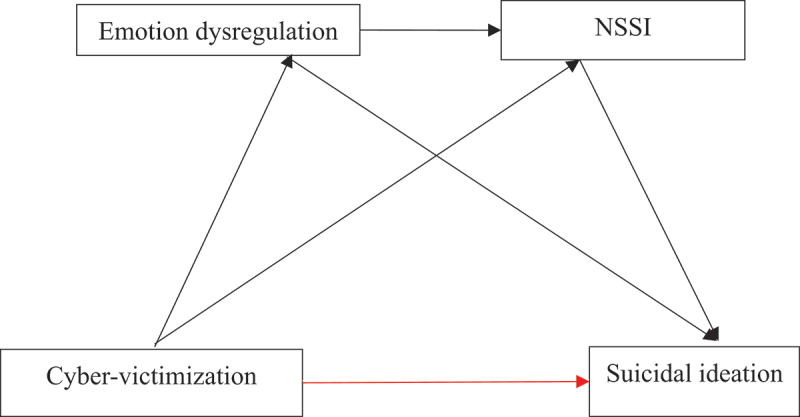
Hypothesized relationships between cyber-victimization, emotion dysregulation, non-suicidal self-injury (NSSI) and suicidal ideation.

In summary, although prior studies have examined the direct association between cyber-victimization and suicidal ideation, or have tested single mediators such as emotion dysregulation or NSSI separately, limited research has simultaneously integrated these variables into a sequential mediation framework. Given that emotion dysregulation may increase vulnerability to NSSI, which in turn elevates suicide ideation risk, examining these mechanisms in a unified chain model may provide a more comprehensive understanding of the psychological pathways linking cyber-victimization to suicidal ideation. The present study extends prior research by testing this sequential mediation model among Chinese college students employing the SPSS PROCESS Model 6. Building on prior empirical research, we have formulated a theoretical hypothesis model (illustrated in Figure 1) and propose the following four hypotheses:

1. Cyber-victimization is positively associated with suicidal ideation among college students.
2. Cyber-victimization is indirectly associated with suicidal ideation in college students through the mediating role of emotion dysregulation.
3. Cyber-victimization is also indirectly associated with suicidal ideation via the mediating role of NSSI.
4. Finally, cyber-victimization is indirectly associated with suicidal ideation through a sequential mediation involving both emotion dysregulation and NSSI.

## Methods

### Recruitment

A cross-sectional study design was employed for our investigation. Data were collected from December 2022 to January 2023 with a convenience sampling approach. The recruited information was shared on a psychological community app designed for college students to communicate and receive expert responses from psychologists. All app users are registered students from a specific university with a focus on science and engineering fields. Participants in activities posted on the app can earn points, which they can later exchange for credits. The recruitment advertisement included details about the research and featured a QR code. Interested students could scan the QR code to access the survey page. Participation was entirely voluntary, and no direct or targeted invitations were sent to individual students. Participants in the survey earn 5 points as a reward. This small, non-monetary incentive was provided solely to acknowledge participation time and was not contingent upon specific responses or completion speed, thereby minimising potential response bias. Students interested in this research could access this notification through websites on their mobile phones or computers. Participants were required to provide informed consent to continue with the questionnaire; those who declined would be directed immediately to the end of the survey. The informed consent form clearly described the study purpose, procedures, voluntary nature of participation, confidentiality measures, and the right to withdraw at any time without penalty. This study utilised an anonymous survey format, ensuring that no private information about the students was collected. Specifically, no personally identifiable information (e.g., names, student identification numbers, or IP addresses) was recorded. The survey platform was configured to prevent multiple submissions from the same account. All questions were mandatory, and participants had to answer every question before submitting the questionnaire. A total of 2106 students accessed the questionnaire. Of these, 8 declined after viewing the consent form, and 2098 provided consent and completed the survey. Students who met the following three criteria were eligible for inclusion in the study:(1) currently enrolled undergraduate students at the university, (2) fluent Chinese speakers able to understand the questionnaire items, and (3) willing to provide informed consent. Exclusion criteria were predefined before data analysis and included: (1) questionnaires completed in less than 3 minutes, based on pilot testing indicating that this duration was insufficient for careful reading and responding, suggesting insufficient engagement; and (2) questionnaires containing a large number of repeated or uniform answers, indicating inattentive responding. Ultimately, 2001 questionnaires were included in the statistical analysis. The study received approval from the Ethics Committee of the University of Electronic Science and Technology of China (106142023010424879). All procedures were conducted in accordance with institutional ethical standards.

### Measures

#### Cyber-victimization

The Revised Cyber Bullying Inventory (RCBI) (Chu & Fan, 2017) comprises two subscales: cyberbullying and cyber-victimization, each containing 14 items. The cyber-victimization subscale was used to evaluate the frequency of individuals’ experiences with cyber-victimization over the past 6 months in the study. A 4-point Likert scale was utilised for scoring, where 1 indicates “never implemented” or “never encountered”, 2 indicates “once”, 3 represents “2–3 times”, and 4 signifies “more than 3 times”. Some examples of the items include: “I was insulted by someone online”. “I was threatened by someone online”. and “I received false information online to defame me”. In this study, the scale demonstrated excellent internal consistency (α = 0.906).

#### Emotion dysregulation

Emotional dysregulation deficits among college students were assessed using the Brief Version of the Difficulties in Emotion Regulation Scale (DERS-16) (Bjureberg et al., 2016). This instrument includes 16 items, each rated on a scale from 1 to 5, where 1 corresponds to “almost never”, 2 indicates “sometimes”, 3 represents “about half the time”, 4 denotes “most of the time”, and five signifies “almost always‘. For example, ’ When I am upset, I start to feel very bad about myself. “ Research conducted by Wang, Guo, and Shen (Wang et al., 2021) has shown that this scale demonstrates strong validity and reliability within the population of Chinese college students. In the present study, the Cronbach’s alpha for the scale was 0.956, indicating excellent internal consistency.

#### Non-suicidal self-injury (NSSI)

The behaviour of NSSI was measured using the Adolescent Non-suicidal Self-injury Assessment Questionnaire, developed by Wan et al (Wan et al., 2018). This scale comprises 12 items designed to assess the frequency of NSSI behaviours occurring, with responses on a Likert-type scale from 0 (Never) to 4 (Always). The questionnaire is divided into two factors: (1) self-injury without obvious tissue damage, referring to behaviours like pinching, scratching, and hair-pulling that do not cause serious injury, and (2) self-injury with obvious tissue damage, such as cutting, burning, or writing symbols on the skin, which can

bleeding or other tissue damage. The severity of NSSI was linked to the total score. In this study, the scale showed excellent internal consistency (α = 0.955).

#### Suicidal ideation

Two questions were asked to evaluate individuals’ suicidal ideation: “Have you ever thought about committing suicide?” and “Have you thought about committing suicide in the past 12 months?” (Shen, 2020). Responses to each question were scored from 1 (never) to 3 (always), with higher scores indicating a greater level of suicidal ideation. The Cronbach’s alpha for this scale in our study was 0.697.

### Statistical analysis

The SPSS 27.0 software was used for the analysis. Descriptive statistics were used to present the participants’ demographic characteristics, and then the data were standardised for the variables. Pearson correlation coefficients were used to examine the correlations between the study variables. The PROCESS macro for SPSS (Model 6) was selected to test the hypothesised serial mediation among observed composite variables, as it provides a parsimonious regression-based bootstrapping approach for estimating indirect effects. A bootstrap test with 5000 repeat samplings was used to test the statistical robustness, with a 95% confidence interval (CI) not containing 0 indicating a significant moderating effect (Hayes, 2018). Gender and age were controlled in the models. A *p*-value < 0.05 was regarded as statistically significant.

Since the data in our study were collected through self-report measures, which may be vulnerable to common method bias, anonymous questionnaires were used. It is specified that the data are for scientific purposes only, aiming to minimise common method bias as much as possible. Additionally, we conducted a Harman single-factor test on all variables using exploratory factor analysis without rotation. This analysis revealed six factors with eigenvalues greater than 1; however, the first factor accounted for only 32.44% of the variance, which is still below the critical threshold of 40% (Podsakoff et al., 2003). While this result does not eliminate common method variance, it indicates that common method bias is unlikely to distort the data interpretation.

Multicollinearity diagnostics revealed that all Variance Inflation Factor (VIF) values ranged from 1.000 to 1.137, which are well below the conservative threshold of 5, indicating no significant concerns about multicollinearity (Shrestha, 2020).

## Results

### Demographic characteristics of the participants

A total of 2001 students aged 18 to 24 participated in our study, with an average age of 18.47 ± 0.89 years. Among these students, there were 1501 males (75.00%) and 500 females (25.00%). The university we surveyed focuses on science and engineering, with a male-to-female ratio of approximately 4:1, which is consistent with the male-to-female ratio in this survey. The distribution across academic years was as follows: freshmen accounted for 76.81% (1,537 students), sophomores made up 17.79% (356 students), juniors comprised 4.30% (86 students), and seniors represented 1.10% (22 students). The proportion of freshmen and sophomores is relatively high, which may be related to the fact that juniors and seniors tend to use the psychological community app less frequently after they have earned their credits.

### Prevalence of suicidal ideation, cyber-victimization, and NSSI

A total of 355 participants reported experiencing suicidal ideation in the past 12 months, which indicates a prevalence rate of 17.74%. Additionally, 744 college students (37.18%) reported engaging in non-suicidal self-injury (NSSI), and 837 students (41.83%) experienced cyber-victimization.

### Correlation between cyber-victimization, suicidal ideation, NSSI, and emotion dysregulation

Pearson correlation analysis was conducted to explore the relationships among all variables. All variables in the model were standardised. Table 1 indicates that all variables are significantly correlated with each other. Cyber-victimization showed significant and positive correlations with emotion dysregulation and NSSI (*r* = 0.250, *p* < 0.001; *r* = 0.562, *p* < 0.001). Emotion dysregulation was significantly and positively correlated with NSSI (*r* = 0.326, *p* < 0.001). Suicidal ideation was significantly positively correlated with cyber-victimization, emotion dysregulation, and NSSI (*r* = 0.210, *p* < 0.001; *r* = 0.378, *p* < 0.001; *r* = 0.308, *p* < 0.001).

**Table 1.** Descriptive statistics and correlation analysis (*N* = 2001).

	*Mean*	*SD*	Cyber-victimization	emotion dysregulation	NNSI	suicidal ideation
Cyber-victimization	16.01	4.60	1			
Emotion dysregulation	34.13	14.21	0.250***	1		
NSSI	2.49	6.16	0.562***	0.326***	1	
Suicidal ideation	0.53	0.83	0.210***	0.378***	0.308***	1

*Note*. SD, standard deviation; NSSI, non-suicidal self-injury; ***p < 0.001.

### Testing for the mediation effect

Cyber-victimization, emotion dysregulation, non-suicidal self-injury (NSSI), and suicidal ideation are significantly correlated, which meets the statistical requirements (Wen & Ye, 2014) for further mediating effect analysis of cyber-victimization and suicidal ideation. After controlling for gender and age, Model 6 in SPSS 27.0 compiled by Hayes was used to analyse the chain mediating effect of emotion dysregulation and non-suicidal self-injury (NSSI) in the relationship between cyber-victimization and suicidal ideation

**Figure 2.**
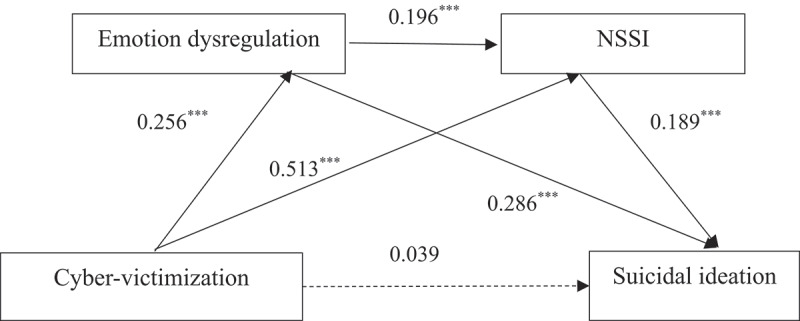
Chain mediation model showing standardized regression coefficients for paths between cyber-victimization, emotion dysregulation, NSSI, and suicidal ideation.

The results of the regression analysis (Table 2) revealed that cyber-victimization had no significant link with suicidal ideation (β = 0.039, *p* > 0.5). Hypothesis 1 is not supported. Cyber-victimization is significantly and positively associated with emotion dysregulation (β = 0.256, *p* < 0.001) and NSSI (β = 0.513, *p* < 0.001) among college students. Emotion dysregulation was found to be positively linked to NSSI (β = 0.196, *p* < 0.001) and suicidal ideation (β = 0.286, *p* < 0.001). Additionally, NSSI positively links to suicidal ideation (β = 0.189, *p* < 0.001). The detailed path model is shown in Figure 2.

**Table 2.** Regression analysis of variable relationship in the chain mediation model.

Regression equation		Fitting index			Significance	
Result variable	Predictor variable	*R*	*R*^*2*^	*F*	*β*	*t*
Emotion dysregulation	cyber-victimization	0.28	0.08	58.72***	0.256	11.89***
NSSI	cyber-victimization	0.59	0.35	271.81***	0.513	27.47***
	emotion dysregulation				0.196	10.41***
Suicidal ideation	cyber-victimization	0.45	0.21	103.88***	0.039	1.62
	emotion dysregulation				0.286	13.40***
	NSSI				0.189	7.61***

*Note*. All the variables in the model have been standardized; NSSI, non-suicidal self-injury; ***p < 0.001.

Table 3 shows that emotion dysregulation and NSSI significantly mediate the relationship between cyber-victimization and suicidal ideation, with a total indirect effect of 0.179, accounting for 81.92% of the total effect. This mediating effect includes three indirect effects:

**Table 3.** The indirect effect of cyber-victimization on suicidal ideation.

		Indirect effects	Boot SE	Boot LLCI	Boot ULCI	Relative mediation effect
Total		0.1794	0.0224	0.14	0.23	81.92%
Cyber-victimization→Emotion dysregulation→Suicidal ideation		0.0732	0.0096	0.06	0.09	33.42%
Cyber-victimization→NSSI→Suicidal ideation		0.0968	0.0185	0.06	0.14	44.20%
Cyber-victimization→Emotion dysregulation→NSSI→Suicidal ideation		0.0094	0.0028	0.005	0.016	4.29%

*Note*. Boot SE, Boot LLCI, and Boot ULCI refer to the standard error and the upper and lower bounds of the 95% confidence intervals of the indirect effects estimated by the bootstrap method, respectively. All variables in the model have been standardized; NSSI: non-suicidal self-injury.

Path 1: Cyber-victimization → Emotion dysregulation → Suicidal ideation (effect = 0.073, 95% CI: 0.06 to 0.09); Path 2: Cyber-victimization → NSSI → Suicidal ideation (effect = 0.097, 95% CI: 0.06 to 0.14); and Path 3: Cyber-victimization → Emotion dysregulation → NSSI → Suicidal ideation (effect = 0.009, 95% CI: 0.005 to 0.016). The ratios of the three indirect effects to the total effect are 33.42%, 44.20%, and 4.29% for paths 1, 2, and 3, respectively. All three indirect effects were statistically significant because their 95% confidence intervals did not include zero. Therefore, Hypotheses 2, 3, and 4 are confirmed.

## Discussion

This study enhances existing research by clarifying the mechanisms between cyber-victimization and suicidal ideation among college students in China. The findings indicate that the experience of cyber-victimization significantly increases the likelihood of suicidal ideation. Specifically, cyber-victimization is indirectly linked to suicidal ideation through the mediating effects of emotion dysregulation (mediation effect 33.42%) and NSSI (mediation effect 44.20%), as well as through the chain mediating effect involving both emotion dysregulation and NSSI (mediation effect 4.29%). By integrating emotion dysregulation and NSSI into a single explanatory framework, the findings provide a more nuanced understanding of how emotional processes may translate interpersonal stress into self-injurious behaviours and suicidal thoughts. The chain mechanism suggests that interventions targeting emotion regulation may not only directly reduce suicidal ideation but also indirectly prevent NSSI, thereby interrupting a broader risk pathway.

The prevalence of suicidal ideation in our study was 17.74%, similar to the existing literature (Lew et al., 2020), which reported a 19.2% prevalence of 12-month suicidal ideation among Chinese college students. Additionally, our study found a prevalence of 41.83% for cyber-victimization among college students, higher than previous studies, which ranged from 7.82% to 22.8% (Jin et al., 2023; Sun et al., 2024). It is plausible that male college students made up a relatively high proportion of our sample. Jin et al. (2023) found that being male among college students was an independent risk factor for cyber-victimization. Compared to the females, male college students tend to be associated with higher impulsivity (Cross et al., 2011) and spend more time playing online games (Li et al., 2018), making them at higher risk of experiencing cyber-victimization. The prevalence rate of NSSI differed significantly between our study (37.15%) and that of Liao et al. (14.97%) (2025). It is possible that the recruitment in our study through an APP aimed at psychological support attracts more people with psychological distress to participate.

Our findings confirmed that cyber-victimization was positively related to suicidal ideation among college students, which is consistent with the results of previous studies (Bai et al., 2021; Nikolaou, 2017; Quintana-Orts et al., 2022). Researchers have found that cyber-victimization can disrupt emotional balance, manifesting as feelings of loneliness, depressive symptoms, perceived stress, and reduced life satisfaction (Estévez et al., 2019). These factors have been recognised, to some extent, as predictors of suicidal ideation. Additionally, individuals who have experienced cyber-victimization tend to engage in greater emotional repression compared to non-victims. In the Chinese cultural context, seeking psychological help is often perceived as indicating “incompetence” or causing a “loss of face”, which may discourage individuals from pursuing mental health support (Vogel et al., 2024). This tendency can exacerbate their internalising behavioural issues, manifesting in outcomes such as non-suicidal self-injury behaviours and suicidal ideation (Brausch et al., 2022; Chu et al., 2022).

The current findings demonstrated the mediating role of emotion dysregulation in cyber-victimization and suicidal ideation, supporting hypothesis 2. This finding aligns with prior research. Estévez et al. (2019) highlighted that individuals who experience cyber-victimization often struggle with emotion dysregulation, which hinders their ability to focus on selecting and implementing positive coping strategies (Nabuzoka et al., 2009). Furthermore, Swee et al. (2020) reported that internal dysfunctional emotion regulation was most strongly associated with suicide ideation, which is consistent with the occurrence of suicide ideation in the three-step theory of suicide (Klonsky & May, 2015). When the psychological pain caused by cyber-victimization cannot be alleviated through emotion regulation strategies, the likelihood of suicidal ideation increases, and this suggests that improving college students’ emotional regulation skills can reduce suicidal ideation. A systematic review recommends group emotion regulation interventions, such as psychoeducation on emotions, strategies for enhancing emotional awareness, identifying and labelling emotions, accepting and tolerating emotions, promoting the appropriate expression of emotions, monitoring emotions and reactions, and understanding emotional triggers, significantly improve individuals’ ability to regulate their emotions (Moore et al., 2022).

The findings confirmed the mediating role of NSSI in the association between cyber-victimization and suicidal ideation, supporting Hypothesis 3. This result is consistent with previous studies showing that cyber-victimization increases the likelihood of engaging in NSSI (Lanzillo et al., 2023; Liao et al., 2025) and that NSSI in turn elevates the risk of suicidal ideation, particularly when it occurs more frequently or at an earlier age (Kiekens et al., 2018). These findings underscore the need to consider not only the occurrence of NSSI but also its frequency and onset age, and further suggest that reducing NSSI behaviours may help to mitigate suicidal ideation.

This study identified the chain mediating roles of emotion dysregulation and NSSI in the relationship between cyber-victimization and suicidal ideation, thereby supporting Hypothesis 4. The model suggests that the effect of cyber-victimization on suicidal ideation among college students may be largely accounted for by difficulties in emotion regulation and the occurrence of NSSI. Prior research has shown that individuals involved in cyber-victimization situations display more impaired emotional functioning and lower emotion regulation abilities compared to those not involved (Cañas et al., 2020). Accordingly, experiences of cyber-victimization may heighten distressing emotions among college students. A meta-analysis further indicated that difficulties in regulating distressing emotional states often underlie NSSI (Taylor et al., 2018), suggesting that individuals with emotion dysregulation are more likely to engage in self-injury. Consistent with this, Brausch and Woods (2019) reported that deficits in emotion regulation and NSSI are significant predictors of suicidal ideation. Taken together, these findings imply that college students who experience cyber-victimization and struggle with emotion dysregulation are particularly vulnerable to NSSI and subsequent suicidal ideation.

This study offers meaningful insights that may inform practices within university student affairs departments. Although directly reducing the incidence of cyber-victimization remains challenging (Lan et al., 2022), the identified mediating roles of emotion dysregulation and NSSI suggest that interventions targeting these factors should be prioritised, particularly for students who experience cyber-victimization. Emotion Regulation Group Therapy (ERGT), which has strong empirical support (Gratz et al., 2015), may be incorporated into campus counselling services to enhance acceptance, commitment, and behavioural control rather than directly attempting to eliminate emotional distress. In addition, web-based peer support interventions have shown promise in reducing NSSI behaviours (Kruzan et al., 2022), offering accessible and scalable support within university settings. Furthermore, mindfulness-based intervention (Zheng et al., 2024) and cognitive reappraisal training (Li et al., 2025) may further strengthen students’ emotion regulation capacities. Mindfulness promotes present-moment awareness, helping individuals move beyond excessive rumination and anticipatory anxiety, while cognitive reappraisal facilitates more adaptive interpretations of stressful events, thereby alleviating negative emotions and reducing psychological risk. Importantly, when implementing these approaches in Chinese university contexts, cultural considerations should not be overlooked. Influenced by Confucian values emphasising endurance and interpersonal harmony, some students may be more likely to suppress emotional expression or hesitate to seek psychological help. Designing culturally sensitive interventions that normalise emotional expression and reduce stigma surrounding help-seeking may enhance their effectiveness. Educational institutions can therefore play a crucial role by increasing awareness of available mental health resources, fostering interpersonal support environments, and adopting culturally responsive, student-centred approaches to intervention.

## Limitation

Several limitations should be acknowledged. Firstly, we acknowledge that the cross-sectional design limits causal and temporal inferences. Future longitudinal or experimental studies are warranted to clarify further the temporal dynamics among cyber-victimization, emotion dysregulation, NSSI, and suicidal ideation. Second, despite using an anonymous online survey with assurances of confidentiality, recall bias may have occurred; reports of cyber-victimization, NSSI, and suicidal ideation could have been inaccurate. Future research could incorporate multi-informant assessments (e.g., peer reports or clinician ratings) to reduce potential shared method variance. Third, the online recruitment strategy may have overrepresented digitally active students, while gender and grade imbalances further reduced the sample’s representativeness. In addition, this study did not consider potential mediators such as specific forms of cyber-victimization (e.g., online rumours, threatening texts or photos) and distinct emotion regulation strategies. Cross-cultural research suggests that emotion regulation strategies exert differential effects on psychological outcomes in Western versus Chinese contexts (Zhou et al., 2020). Future research should refine sampling methods, adopt qualitative or mixed-methods approaches, and further examine the mediating roles of specific emotion regulation strategies (e.g., expressive suppression) in the relationship between cyber-victimization and suicidal ideation.

## Conclusion

Our findings suggest that screening and interventions targeting emotion regulation and NSSI, such as group emotion skills training, peer-support interventions, mindfulness interventions, or cognitive reappraisal training, can be integrated into university mental health services. Students who experience cyber-victimization appear to be a high-risk group who may particularly benefit from these targeted interventions, highlighting the practical value of proactively supporting these students to reduce suicidal ideation.

## Data Availability

The data that support the findings of this study are available from the corresponding authors upon reasonable request.
